# Prognostic value of Ki-67 in nasopharyngeal carcinoma: a meta-analysis

**DOI:** 10.1042/BSR20203334

**Published:** 2021-05-06

**Authors:** Yulin Li, Liang Yue, Yanqing Li, Qinxiu Zhang, Xin Liang

**Affiliations:** 1 School of Medicine and Life Sciences, Chengdu University of Traditional Chinese Medicine, Chengdu, Sichuan 611137, China; 2 Department of Otorhinolaryngology, Hospital of Chengdu University of Traditional Chinese Medicine, Chengdu, Sichuan 610075, China

**Keywords:** evidence-based medicine, Ki-67, meta-analysis, nasopharyngeal carcinoma, prognostic factors

## Abstract

The prognostic value of Ki-67 in nasopharyngeal carcinoma (NPC) was controversial according to previous studies. We aimed to clarify the association between K-67 expression and survival in NPC through meta-analysis. We conducted a meta-analysis to explore the potential prognostic effect of Ki-67 on overall survival (OS), disease-free survival (DFS), distant metastasis-free survival (DMFS), and local recurrence-free survival (LRFS) in NPC. A total of 13 studies comprising 1314 NPC patients were included. High Ki-67 expression was associated with poor OS (hazard ratio [HR]= 2.70, 95% confidence interval [CI]= 1.97–3.71, *P*<0.001), DFS (HR = 1.93, 95% CI = 1.49–2.50, *P*<0.001), and LRFS (HR = 1.86, 95% CI = 1.11–3.12, *P*=0.019). However, there was no significant association between Ki-67 and DMFS (HR = 1.37, 95% CI = 0.78–2.38, *P*=0.270). Furthermore, the prognostic role of Ki-67 was maintained throughout different sample sizes, analyses of HR, and study designs for OS and DFS in various subgroups. Elevated Ki-67 expression is a reliable prognostic factor for poorer survival outcomes in NPC.

## Introduction

Nasopharyngeal carcinoma (NPC) is a rare cancer that originates from the lining of the nasopharynx [1]. The incidence of NPC is distinguished geographically; it is relatively high in Southeast Asia but low in Western countries [2]. The management of NPC chiefly depends on the disease status. For non-metastatic disease, radiotherapy is the mainstay treatment strategy [3]. For metastatic and locally recurrent disease, chemoradiotherapy and systemic therapies are the current therapeutic modalities [4]. However, over 20% of patients with NPC develop distant metastasis or recurrence after initial treatment, resulting in a poor prognosis [5]. Prognostic markers, such as tumor-node-metastasis (TNM) staging system and Epstein–Barr viral (EBV) DNA load, are widely used for prognostication and are required for the clinical management of patients with NPC. However, these parameters do not provide adequate prognostic information for individual patients. Therefore, there is an urgent need to develop valid prognostic factors for NPC.

Sustaining proliferative signaling is a hallmark of cancer cells, and tumor proliferation markers can provide a prognosis for patients [6]. Ki-67 is one of the most common proliferation markers [7] which can be detected in the cell nuclei during all phases of the cell cycle (G_1_, S, G_2_, and mitosis) [8]. Ki-67 has been widely investigated as a prognostic indicator in various cancers, including non-muscle invasive bladder [8], ovarian [9], gastric [10], breast [11], and non-small cell lung cancer [12]. A variety of studies reported the prognostic value of Ki-67 in patients with NPC; however, the results were inconsistent [13–15]. Therefore, we comprehensively and systematically searched for eligible studies to clarify the prognostic role of Ki-67 in patients with NPC.

## Materials and methods

### Study guidelines and ethics

We performed the present meta-analysis in accordance with the Preferred Reporting Items for Systematic Reviews and Meta-Analyses guidelines [16]. Ethical approval was not necessary because the present study did not involve patient consent.

### Literature search

The literature search was conducted from the inception of the present study to 19 June 2020. We retrieved the electronic databases of PubMed, Web of Science, Embase, Scopus, and The Cochrane Library. The following search terms were used: ‘Ki-67,’ ‘Ki67,’ ‘MIB-1,’ ‘prognosis,’ ‘prognostic,’ ‘survival,’ ‘outcome,’ ‘nasopharyngeal carcinoma,’ ‘nasopharyngeal cancer,’ and ‘nasopharyngeal neoplasms.’ We manually examined the reference lists of relevant literature to identify eligible studies.

### Selection criteria

The inclusion criteria were as follows: (1) studies evaluating the association between Ki-67 expression and survival in patients with NPC; (2) Ki-67 detection in tumor tissue using immunohistochemistry (IHC); (3) hazard ratios (HRs) and 95% confidence intervals (CIs) for survival outcomes were provided in text or could be calculated; (4) a cutoff value was identified to stratify high and low Ki-67 expression; (5) published in English. Studies that did not meet all the inclusion criteria were excluded. Two reviewers (Y.l.L. and L.Y.) independently evaluated candidate studies, and all disagreements were resolved by consensus.

### Data extraction

Two investigators (Y.l.L. and L.Y.) extracted the data of the eligible studies independently with a predefined form. All discrepancies were resolved by discussion with a third investigator (X.L.). Extracted data included the name of the author, year of publication, study location, survival outcomes, TNM stage, treatment method, sample size, study design, and analysis of HR. Overall survival (OS) was the primary endpoint. Disease-free survival (DFS), distant metastasis-free survival (DMFS), and local recurrence-free survival (LRFS) were secondary endpoints.

### Quality assessment

The Newcastle–Ottawa Scale (NOS) [17] was employed to assess the quality of the methodology used in the included studies. It contains three domains: selection of patients (0–4 points), comparability of cohorts (0–2 points), and outcome assessment (0–3 points). NOS scores of at least 6 were considered high quality.

### Statistical analysis

The association between Ki-67 and OS, DFS, DMFS, and LRFS was evaluated by combining HRs and 95% CIs of included studies. HR > 1 without a 95% CI overlapping 1 indicated that overexpression of Ki-67 was the prognostic risk factor, and HR < 1 without a 95% CI overlapping 1 was a protective factor. Statistical heterogeneity was calculated according to Higgins *I*^2^ statistic and Cochran’s Q test. The *I*^2^ values > 50% or *P* of heterogeneity < 0.1 were considered significant heterogeneity, and consequently the random-effects model was adopted; if not, the fixed-effects model was selected. We performed subgroup analyses stratified by clinical variables including geographical region, TNM stage, treatment, sample size, cutoff value, analysis of HR, and study design for OS and DFS. Publication bias was detected using Begg’s rank correlation test and Egger’s linear regression test. Stata statistical software (version 12.0; Stata Corp, College Station, TX, U.S.A.) was used to analyze the extracted data. A *P*-value <0.05 was considered significant.

## Results

### Study search

**Figure 1:**
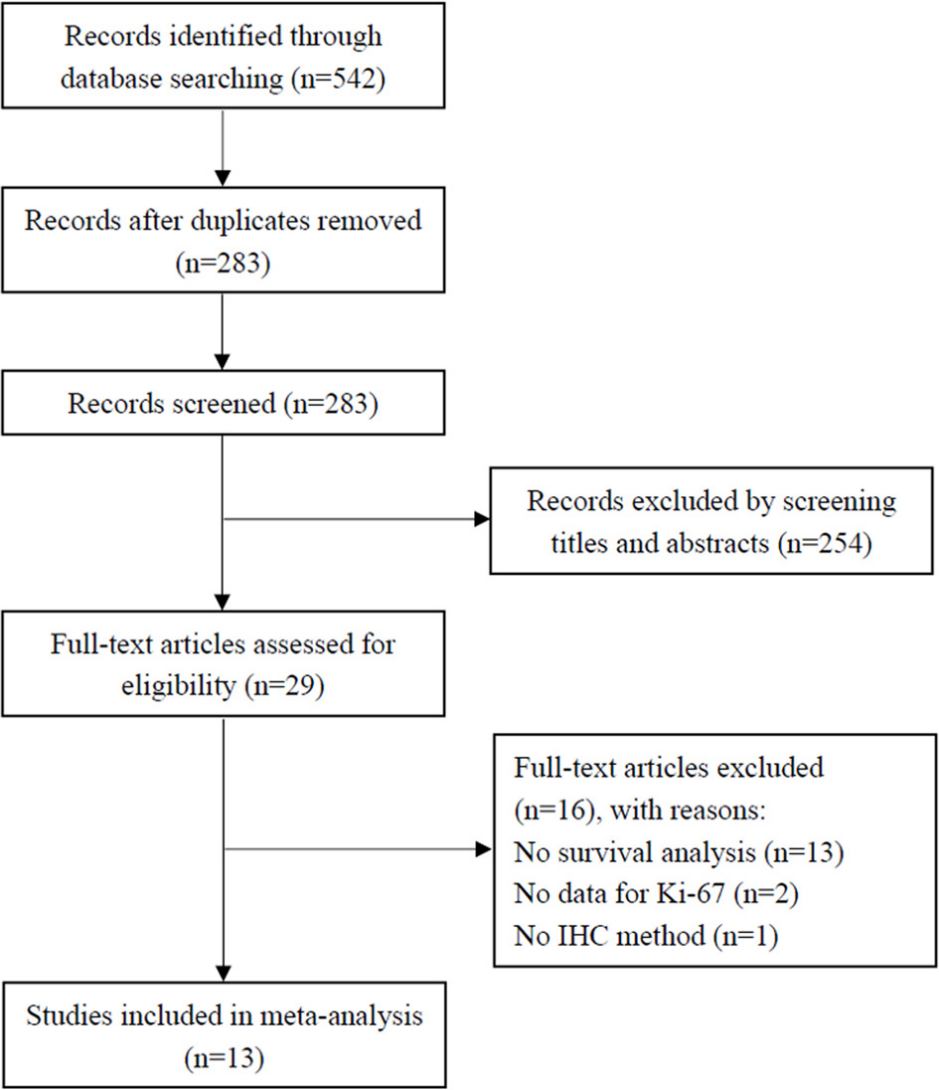
Flow diagram of the literature identification process

Initially, 542 studies were retrieved from the databases, and 283 studies remained after duplicates were removed. By examining titles and abstracts, 254 studies were discarded, leaving 29 studies for full-text evaluation. Sixteen studies were excluded for the following reasons: no survival analysis (*n*=13), no data for Ki-67 (*n*=2), and no IHC method (*n*=1). Finally, 13 studies that met the inclusion criteria were included in the present meta-analysis [13–15,18–27] (Figure 1).

### Characteristics of included studies

The included studies were published between 2000 and 2019 (Table 1). The studies were conducted in six countries/regions, including China (*n*=8) [19,14,22–27], Tunisia (*n*=1) [13], Taiwan (*n*=1) [18], Greece (*n*=1) [20], Turkey (*n*=1) [21], and Japan (*n*=1) [15]. Two studies [19,20] were prospective trials, and eleven studies [13–15,18,21–27] had a retrospective study design. Nine studies [13,14,18,21–26] recruited patients with TNM stages I–IV, two studies [19,27] with TNM stages III–IV, and two studies [20,15] with TNM stages II–IV. A total of 1314 patients were included in the meta-analysis. Five studies provided multivariate HRs [13,14,20,26,27], and eight studies presented univariate HRs [15,18,19,21–25]. The cutoff values of Ki-67 were not uniform between eligible studies: ≥10% (*n*=4) [14,21,22,27], ≥5% (*n*=3) [13,19,20], ≥50% (*n*=3) [15,23,24], ≥25% (*n*=1) [25], ≥77.5% (*n*=1) [26], and H-score ≥ median (*n*=1) [18]. NOS scores of included studies were no less than 6 (high quality), and details for each study are listed in Table 2.

**Table 1:** Baseline characteristics of studies included in the present meta-analysis

Study	Year	Country/ region	Outcome	TNM stage	Treatment	Sample size	Cut-off value for Ki-67	Analysis of HR	Study design	Detection method	NOS score
Ben-Haj-Ayed	2016	Tunisia	OS, DFS	I–IV	Mixed	71	≥5%	Multivariate	Retrospective	IHC	7
Chang	2017	Taiwan	DFS, DMFS, LRFS	I–IV	Mixed	124	H-score ≥ median	Univariate	Retrospective	IHC	7
Fan	2019	China	DMFS	III–IV	Chemoradiotherapy	147	≥5%	Univariate	Prospective	IHC	8
Fountzilas	2012	Greece	OS, DFS	II–IV	Chemoradiotherapy	141	≥5%	Multivariate	Prospective	IHC	9
Genç	2000	Turkey	OS	I–IV	Radiotherapy	35	≥10%	Univariate	Retrospective	IHC	6
Guan	2015	China	OS, DFS	I–IV	Mixed	58	≥10%	Multivariate	Retrospective	IHC	7
Kijima	2001	Japan	OS	II–IV	Radiotherapy	19	≥50%	Univariate	Retrospective	IHC	6
Lu	2017	China	OS, DFS, DMFS, LRFS	I–IV	Chemoradiotherapy	334	≥10%	Univariate	Retrospective	IHC	8
Shi	2015	China	OS	I–IV	Chemoradiotherapy	55	≥50%	Univariate	Retrospective	IHC	7
You	2015	China	OS	I–IV	Mixed	118	≥50%	Univariate	Retrospective	IHC	7
Zhang	2016	China	OS	I–IV	Chemoradiotherapy	59	≥25%	Univariate	Retrospective	IHC	8
Zhao	2018	China	OS, DFS	I–IV	Mixed	45	≥77.5%	Multivariate	Retrospective	IHC	6
Zhao	2017	China	DFS	III–IV	Mixed	108	≥10%	Multivariate	Retrospective	IHC	8

**Table 2:** Details of NOS scores for studies included in this meta-analysis

Study	Year	Selection				Comparability	Outcome			NOS score
		Representativeness of the exposed cohort	Selection of the non- exposed cohort	Ascertainment of exposure	Demonstration that outcome of interest was not present at start of study	Comparability of cohorts on the basis of the design or analysis	Assessment of outcome	Follow-up long enough for outcomes to occur	Adequacy of follow-up of cohorts	
Ben-Haj-Ayed	2016	★	★	★	★	★	★	-	★	7
Chang	2017	★	★	★	★	★	★	★	-	7
Fan	2019	★	★	★	★	★★	★	★	-	8
Fountzilas	2012	★	★	★	★	★★	★	★	★	9
Genç	2000	★	★	-	★	★	★	★	-	6
Guan	2015	★	★	★	★	★	★	-	★	7
Kijima	2001	★	★	★	★	★	★	-	-	6
Lu	2017	★	★	★	★	★★	★	-	★	8
Shi	2015	★	★	★	★	★	★	★	-	7
You	2015	★	★	★	★	★	★	★	-	7
Zhang	2016	★	★	★	★	★★	★	★	-	8
Zhao	2018	★	★	★	-	★	★	★	-	6
Zhao	2017	★	★	★	★	★★	★	-	★	8

### Prognostic value of Ki-67 for survival outcomes

**Figure 2:**
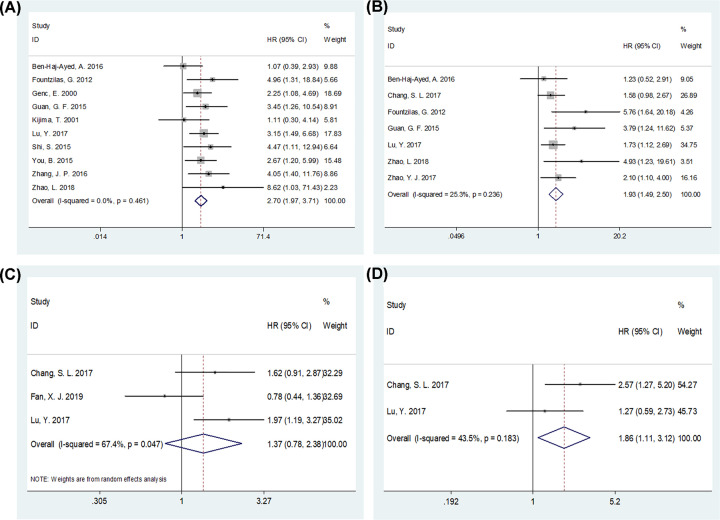
The forest plots depicting the prognostic value of Ki-67 for NPC Forest plots for the relationship between Ki-67 expression and (**A**) OS, (**B**) DFS, (**C**) DMFS, and (**D**) LRFS in patients with NPC.

The prognostic value of Ki-67 for various survival outcomes, including OS, DFS, DMFS, and LRFS, were analyzed. Ten studies with 935 patients [13–15,20–26] provided HRs and 95% CIs for OS (Figure 2A and Table 3). The pooled results were HR = 2.70, 95% CI = 1.97–3.71, and *P*<0.001, suggesting that Ki-67 overexpression was associated with poorer OS in NPC. Data from seven studies with 881 patients [13,14,18,20,22,26,27] were aggregated, and the results were HR = 1.93, 95% CI = 1.49–2.50, and *P*<0.001, which demonstrated the significant prognostic role of Ki-67 in DFS (Figure 2B and Table 2). The correlation between Ki-67 and poor LRFS was also significant (*n*=2, HR = 1.86, 95% CI = 1.11–3.12, *P*=0.019; Figure 2D and Table 3). However, there was no significant association between Ki-67 and DMFS (*n*=3, HR = 1.37, 95% CI = 0.78–2.38, *P*=0.270; Figure 2C and Table 3).

**Table 3:** Summary of the subgroup analysis

Subgroups	Studies (*n*)	Patients (*n*)	Effects model	HR (95% CI)	*P*	Heterogeneity	
						*I*^2^ (%)	*P*
**OS**							
Total	10	935	FEM	2.70 (1.97–3.71)	<0.001	0	0.461
Geographical region							
Asia	8	723	FEM	2.89 (2.05–4.08)	<0.001	0	0.713
Non-Asia	2	212	REM	2.16 (0.48–9.66)	0.314	69.1	0.072
TNM stage							
I–IV	8	775	FEM	2.76 (1.97–3.86)	<0.001	0	0.518
II–IV	2	160	REM	2.34 (0.54–10.13)	0.256	59.2	0.117
Treatment							
Radiotherapy	2	54	FEM	1.90 (1.00–3.61)	0.049	0	0.358
Chemoradiotherapy	4	589	FEM	3.79 (2.28–6.29)	<0.001	0	0.925
Mixed	4	292	FEM	2.39 (1.41–4.03)	0.001	31.5	0.223
Sample size							
<100	7	342	FEM	2.45 (1.63–3.67)	<0.001	20.7	0.272
≥100	3	593	FEM	3.16 (1.90–5.24)	<0.001	0	0.739
Cutoff value							
≥5%	2	212	REM	2.16 (0.48–9.66)	0.314	69.1	0.072
≥10%	3	427	FEM	2.79 (1.75–4.47)	<0.001	0	0.747
≥50%	3	192	FEM	2.52 (1.38–4.59)	0.003	14.8	0.309
Others	2	104	FEM	4.72 (1.82–12.23)	0.001	0	0.533
Analysis of HR							
Univariate	6	620	FEM	2.74 (1.89–3.97)	<0.001	0	0.646
Multivariate	4	315	FEM	2.61 (1.41–4.82)	0.002	44.2	0.146
Study design							
Retrospective	9	794	FEM	2.61 (1.88–3.61)	<0.001	0	0.443
Prospective	1	141	-	4.96 (1.31–18.81)	0.019	-	-
**DFS**							
Total	7	881	FEM	1.93 (1.49–2.50)	<0.001	25.3	0.236
Geographical region							
Asia	5	669	FEM	1.91 (1.45–2.53)	<0.001	1.5	0.398
Non-Asia	2	212	REM	2.48 (0.55–11.19)	0.237	74.7	0.047
TNM stage							
I–IV	5	632	FEM	1.78 (1.33–2.39)	<0.001	16.2	0.312
II–IV/III–IV	2	249	FEM	2.59 (1.46–4.60)	0.001	49.2	0.161
Treatment							
Chemoradiotherapy	2	475	REM	2.72 (0.87–8.50)	0.085	68.1	0.077
Mixed	5	406	FEM	1.89 (1.36–2.64)	<0.001	17.9	0.300
Sample size							
<100	3	174	FEM	2.26 (1.23–4.17)	0.009	49.5	0.138
≥100	4	707	FEM	1.86 (1.40–2.47)	<0.001	20.0	0.290
Cutoff value							
≥5%	2	212	REM	2.48 (0.55–11.19)	0.237	74.7	0.047
≥10%	3	500	FEM	1.97 (1.40–2.79)	<0.001	0	0.433
Others	2	169	REM	2.31 (0.81-6.58)	0.118	56.4	0.130
Analysis of HR							
Univariate	2	458	FEM	1.67 (1.20–2.32)	0.002	0	0.787
Multivariate	5	423	FEM	2.43 (1.60–3.69)	<0.001	33.6	0.197
Study design							
Retrospective	6	740	FEM	1.83 (1.41–2.39)	<0.001	0	0.419
Prospective	1	141	-	5.76 (1.64–20.21)	0.006	-	-
**DMFS**							
Total	3	605	REM	1.37 (0.78–2.38)	0.270	67.4	0.047
**LRFS**							
Total	2	458	FEM	1.86 (1.11–3.12)	0.019	43.5	0.183

### Subgroup analysis

Subgroup analysis for OS and DFS was carried out to investigate the source of heterogeneity. We used seven variables for subgroup analysis, including geographical region, TNM stage, treatment, sample size, cutoff value, analysis of HR, and study design. High Ki-67 expression remained a significant prognostic factor for OS irrespective of treatment, sample size, analysis of HR, and study design (Table 3; all *P*<0.05). In addition, Ki-67 overexpression was associated with poor OS in Asian patients (*P*<0.001), in patients with TNM stage I–IV (*P*<0.001), and with cutoff values ≥10% (*P*<0.001) and ≥50% (*P*=0.003) (Table 3). Elevated Ki-67 expression was predictive of poor DFS in all subgroups of TNM stage, sample size, analysis of HR, and study design (Table 3; all *P*<0.05). Moreover, high Ki-67 expression was correlated with poor DFS in Asian patients (*P*<0.001), in patients receiving mixed treatments (*P*<0.001), and with a Ki-67 cutoff value of ≥10% (*P*<0.001) (Table 3).

### Publication bias

**Figure 3:**
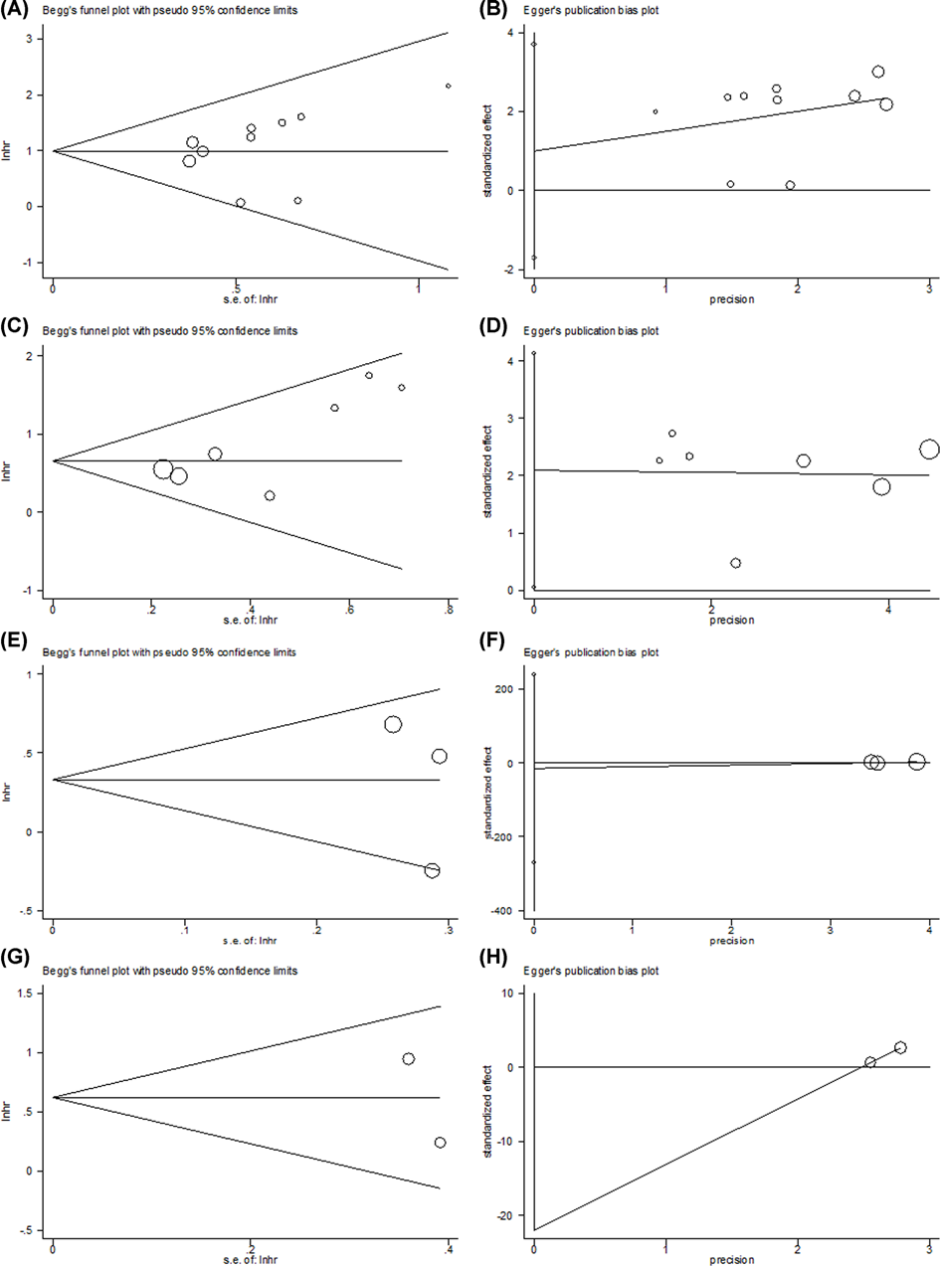
Publication bias test through Begg’s funnel plot and Egger’s regression test in this meta-analysis (**A**) Begg’s test for OS, *P*=0.072. (**B**) Egger’s test for OS, *P*=0.419. (**C**) Begg’s test for DFS, *P*=0.133. (**D**) Egger’s test for DFS, *P*=0.086. (**E**) Begg’s test for DMFS, *P*=0.602. (**F**) Egger’s test for DMFS, *P*=0.591. (**G**) Begg’s test for LRFS, *P*=0.317. (**H**) Egger’s test for LRFS, *P*=1.

The funnel plots of Begg’ test and Egger’s regression test for the meta-analysis are shown in Figure 3. The funnel plots were visually symmetrical, and Egger’s test suggested non-significant publication bias in this meta-analysis.

## Discussion

To our knowledge, the present study is the first meta-analysis exploring the prognostic value of Ki-67 in patients with NPC. The prognostic effect of Ki-67 in patients with NPC is controversial based on the results of relevant studies [13–15,18–27]. The current meta-analysis incorporated data from 13 studies comprising 1314 patients and demonstrated that elevated Ki-67 expression was associated with long-term (OS and DFS) and short-term (LRFS) survival outcomes in patients with NPC. Furthermore, the prognostic role of Ki-67 was maintained throughout different sample sizes, analyses of HR, and study designs for OS and DFS in various subgroups. Ki-67 exerts significant prognostic value for Asian patients, and a Ki-67 cutoff value ≥10% showed consistent prognostic efficiency. According to these results, Ki-67 could be used as a reliable prognostic indicator for NPC, particularly in patients of Asian ethnicity.

Ki-67 is a nuclear protein expressed throughout the cell cycle in proliferating cells that has been investigated as a prognostic marker in various cancers [28,8,12]. The current meta-analysis demonstrated the prognostic role of Ki-67 expression in patients with NPC. Notably, a recent meta-analysis explored the prognostic value of hematological parameters in patients with NPC, which included 23 studies comprising 23417 patients and found neutrophil-to-lymphocyte ratio, C-reactive protein-to-albumin ratio, lymphocyte-to-monocyte ratio, plasma fibrinogen level, and Glasgow prognostic score (GPS) to have an impact on prognostication in NPC [29]. That meta-analysis [29] included 23 studies encompassing 23417 patients and demonstrated a series of hematological indexes, including neutrophil-to-lymphocyte ratio, C-reactive protein-to-albumin ratio, lymphocyte-to-monocyte ratio, plasma fibrinogen level, and GPS have impact on prognostication in NPC. Serum-based parameters are easily accessible and cost-effective in clinical practice. Compared with hematological indexes, Ki-67 has several advantages. First, Ki-67 is stable and cannot be significantly affected by the immunological status of patients; whereas Ki-67 is measured using IHC in tumor tissue, hematological markers are derived from blood-based indexes that can be influenced by chronic inflammation and nutritional condition, not just by cancer. Second, Ki-67 protein, a tumor proliferation marker, is comparable in other types of cancers such as head and neck [30], colorectal [31], and non-small cell lung cancer [12].

The cutoff values of Ki-67 to stratify high and low expression were not consistent in previous studies. In the current meta-analysis, a Ki-67 cutoff value ≥10% showed a consistent prognostic effect. In a recent study on colorectal cancer, a cutoff value of 25% for Ki-67 expression was a good classification tool in the AJCC-8 (American Joint Committee on Cancer 8 edition) stratification [31]. Another study indicated that a Ki-67 index of 5% is better than 2% in stratifying G1 and G2 pancreatic neuroendocrine tumors [32]. These studies suggest that the optimal cutoff value of Ki-67 may vary among different solid tumors. As suggested by our meta-analysis, a cutoff of 10% for Ki-67 expression should be validated for NPC in clinical practice.

The limitations of our meta-analysis need to be acknowledged. First, most of the included studies were retrospective, and heterogeneity may have been introduced. Second, some HRs and 95% CIs extracted using the Kaplan–Meier curves were not directly reported in text; therefore, data calculated may not be accurate. Third, the sample sizes for DMFS/LRFS were relatively small, which may compromise the validity of the prognostic significance of Ki-67 for DMFS and LRFS.

## Conclusions

In summary, elevated Ki-67 expression is a reliable prognostic factor for poorer survival outcomes in NPC. The prognostic effect of Ki-67 remains stable across different subgroups of patients. Therefore, the Ki-67 index may be an important supplementary tool for the prognosis of patients with NPC.

## Data Availability

All data associated with the present study are included in this published article or are available from the corresponding author on reasonable request.

## Abbreviations

CI: confidence interval
DFS: disease-free survival
DMFS: distant metastasis-free survival
HR: hazard ratio
IHC: immunohistochemistry
LRFS: local recurrence-free survival
NOS: Newcastle–Ottawa Scale
NPC: nasopharyngeal carcinoma
OS: overall survival
TNM: tumor-node-metastasis
